# High-performance, inexpensive setup for simultaneous multisite recording of electrophysiological signals and mesoscale voltage imaging in the mouse cortex

**DOI:** 10.1117/1.NPh.5.2.025005

**Published:** 2018-03-29

**Authors:** Edgar Bermudez-Contreras, Sergey Chekhov, Jianjun Sun, Jennifer Tarnowsky, Bruce L. McNaughton, Majid H. Mohajerani

**Affiliations:** a University of Lethbridge, Canadian Centre for Behavioural Neuroscience, Department of Neuroscience, Lethbridge, Alberta, Canada; b University of California at Irvine, Center for the Neurobiology of Learning and Memory, Department of Neurobiology and Behavior, Irvine, California, United States

**Keywords:** microelectrode array, voltage-sensitive dye, mesoscale optical imaging, sensory-evoked and spontaneous activity

## Abstract

Simultaneous recording of optical and electrophysiological signals from multiple cortical areas may provide crucial information to expand our understanding of cortical function. However, the insertion of multiple electrodes into the brain may compromise optical imaging by both restricting the field of view and interfering with the approaches used to stabilize the specimen. Existing methods that combine electrophysiological recording and optical imaging *in vivo* implement either multiple surface electrodes, silicon probes, or a single electrode for deeper recordings. To address such limitation, we built a microelectrode array (hyperdrive, patent US5928143 A) compatible with wide-field imaging that allows insertion of up to 12 probes into a large brain area (8 mm diameter). The hyperdrive is comprised of a circle of individual microdrives where probes are positioned at an angle leaving a large brain area unobstructed for wide-field imaging. Multiple tetrodes and voltage-sensitive dye imaging were used for acute simultaneous registration of spontaneous and evoked cortical activity in anesthetized mice. The electrophysiological signals were used to extract local field potential (LFP) traces, multiunit, and single-unit spiking activity. To demonstrate our approach, we compared LFP and VSD signals over multiple regions of the cortex and analyzed the relationship between single-unit and global cortical population activities. The study of the interactions between cortical activity at local and global scales, such as the one presented in this work, can help to expand our knowledge of brain function.

## 1 Introduction

A fundamental goal of neuroscience is to understand the underlying mechanisms that are employed by the brain to process information. However, in a complex system such as the brain, it is difficult to explain the behavior of the system by only studying its components in isolation. Rather, it is crucial to understand how the interactions of the components give rise to the behavior of the system. Analogously, in order to understand the brain’s dynamics, it is necessary to analyze its activity at different scales. As we know, the behavior of a neuronal network is not only determined by its connection weights but also by the external inputs, which might involve multiple and distant networks. Therefore, in order to fully understand how the brain processes information, it is necessary to be able to study neuronal activity at local and global spatiotemporal scales.^1^^–^^7^

Electrophysiological recordings have been used extensively to study neuronal activity, and with the development of tetrode arrangements,^8^^,^^9^ this method has become an invaluable tool to monitor spiking activity of individual cells at any brain depth. The overall reliability of the technique^10^^–^^13^ provides easy transfer of acquired experimental data into scientific knowledge; however, technical problems that occur when implanting highly dense electrode arrays or intrinsic difficulties in determining the signal sources make this technique difficult to apply for recordings over large areas of the cortex.^14^^–^^16^

Some optical methods, however, offer excellent temporal and spatial resolution for real-time analysis of brain processing.^17^^,^^18^ Wide-field optical imaging and, in particular, voltage-sensitive dye imaging technology has evolved into a convenient tool to study neuronal activity dynamics over large areas of the cortex with high temporal and spatial resolution. The temporal resolution is at the level of millisecond that is on par with electrophysiological recordings.^19^^–^^23^ The spatial resolution reaches 25 to 65  μm per pixel, and the size of imaged brain area is sufficient to record the signs of electrical activity over much of the dorsal mouse cortex,^24^ $1\;{\mathrm{cm}}^{2}$ area monkey’s cortex,^25^ or $0.3\;{\mathrm{cm}}^{2}$ area over the cat cortex.^26^ With these characteristics, voltage-sensitive dye imaging (VSDI) is an excellent technique to study neuronal dynamics over large cortical areas. However, VSDI has limitations that are important for a complete study of the complex interactions of neuronal networks. In particular, VSDI mainly captures subthreshold neuronal activity located within superficial cortical layers.^19^^,^^27^^,^^28^ Therefore, wide-field optical imaging and VSDI, in particular, are good candidates to be combined with multisite electrophysiological recordings. Other recently developed longitudinal mesoscale imaging options such as calcium (GCaMP) or glutamate sensors (iGluSnFR), which reflect neuronal activity at the population level can also be employed for similar purposes.^29^

The idea of combining electrophysiological recordings with VSDI is not new. There are already approaches available to image with parallel cell recordings from acute brain slices.^30^^–^^34^ However, such studies are practically limited to slice preparations. Additionally, simultaneous single-unit recordings and VSDI *in vivo* have been pioneered by Grinvald’s group^18^^,^^19^ and also performed by others with similar techniques,^21^^,^^35^^,^^36^ but their work is limited to a single region in the brain. For a more detailed review of the validity and comparison of VSD with intracellular recordings see Refs. 19 and 21. There have also been approaches combining VSDI over large cortical areas and electrophysiological recordings^20^^,^^30^^,^^37^ but, in these cases, they employ a small number of surface or pipette electrodes that are not suitable for recording signals from deep brain structures or from multiple units. The attempts to combine VSDI with deep recordings *in vivo* include development studies in the newborn rat barrel cortex and thalamus with simultaneous silicon probe recordings and VSDI.^38^^,^^39^ Moreover, combined electrophysiology and VSDI experiments have been carried out on a very well-studied model, primate V1, with separate preparations for each method,^40^ as well as whole-cell recordings with simultaneous VSDI to describe the propagation of excitation in the rat barrel cortex.^21^^,^^41^ An approach to combine mesoscale VSD imaging with deep recordings of extracellular electric potentials at multiple areas in the rodent cortex, however, remained to be developed. Recently, a transparent multielectrode array that registers field potentials from the brain surface *in vivo* can be combined with brain imaging^42^ but that approach is unsuitable for deep multiple single-unit recordings.

In this article, we present a methodology to monitor neuronal activity simultaneously at two different spatial scales and of a different nature. We record local cortical activity over multiple areas using multisite electrodes and global cortical activity using wide-field VSD imaging. To demonstrate our approach, we compare brain activity simultaneously recorded by VSD imaging with local field potentials (LFP) and single-unit spiking activity (SUA) and study their relationship during spontaneous and sensory-evoked activity periods. The study of how these signals interact, as facilitated by our hyperdrive (patent US5928143 A), can potentially expand our understanding of information processing at micro- and mesoscales, which in turn is crucial to study brain function.

## 2 Materials and Methods

### 2.1 Animals

All experiments were carried out on adult (20 to 30 g, age 2 to 4 month) wild-type C57/Bl6 mice (n=5) or B6.Cg-Tg (Thy1-COP4/EYFP) 18Gfng/J mice (n=2). Mice were housed under standard conditions, in clear plastic cages under 12 h light and 12 h dark cycles. Mice were given *ad libitum* access to water and standard laboratory mouse diet at all times. All protocols were approved by the Animal Welfare Committee of the University of Lethbridge and were in accordance with guidelines set forth by the Canadian Council for Animal Care.

### 2.2 Surgery

Mice were anesthetized with 15% urethane (1250  mg/kg) and fixed in a stereotactic apparatus. Body temperature was maintained at 37°C with an electric heating pad regulated by a feedback thermistor. Mice were given dexamethasone (80  μg) intramuscularly to prevent inflammation and lidocaine (50  μl, at 2%) into the area of the skin incision over the skull. The plastic headplate (inner diameter 8 mm) was attached to the bone with dental cement.^43^ An ∼8-mm-diameter single cranial window was made over both cortical hemispheres (2.5 to 5.5 mm, anterior-posterior and 0 to 4 mm laterally from bregma) using a high-speed dental drill.^44^ To keep the brain cool, the drilling was done intermittently and the skull was moistened with artificial CSF composed of NaCl (3.94g), KCl (0.2 g), ${\mathrm{MgCl}}_{2}$ $6H_{2}O$ (0.102 g), ${\mathrm{CaCl}}_{2}$ $2H_{2}O$ (0.132 g), and Na HEPES (0.651 g) in 500 ml of ultrapure Milli-Q water. Caution was taken to keep the dura intact when removing the bone. Once the bone was removed, dura mater was also carefully removed as described previously.^44^ For each hour under anesthesia, the mouse was given 10  ml/kg of 20 mM glucose in brain buffer IP to maintain hydration. In order to alleviate respiratory distress induced by urethane anesthesia, a tracheotomy was performed to allow intubation to maintain airways open without the need of artificial ventilation.^45^

**Fig. 1:**
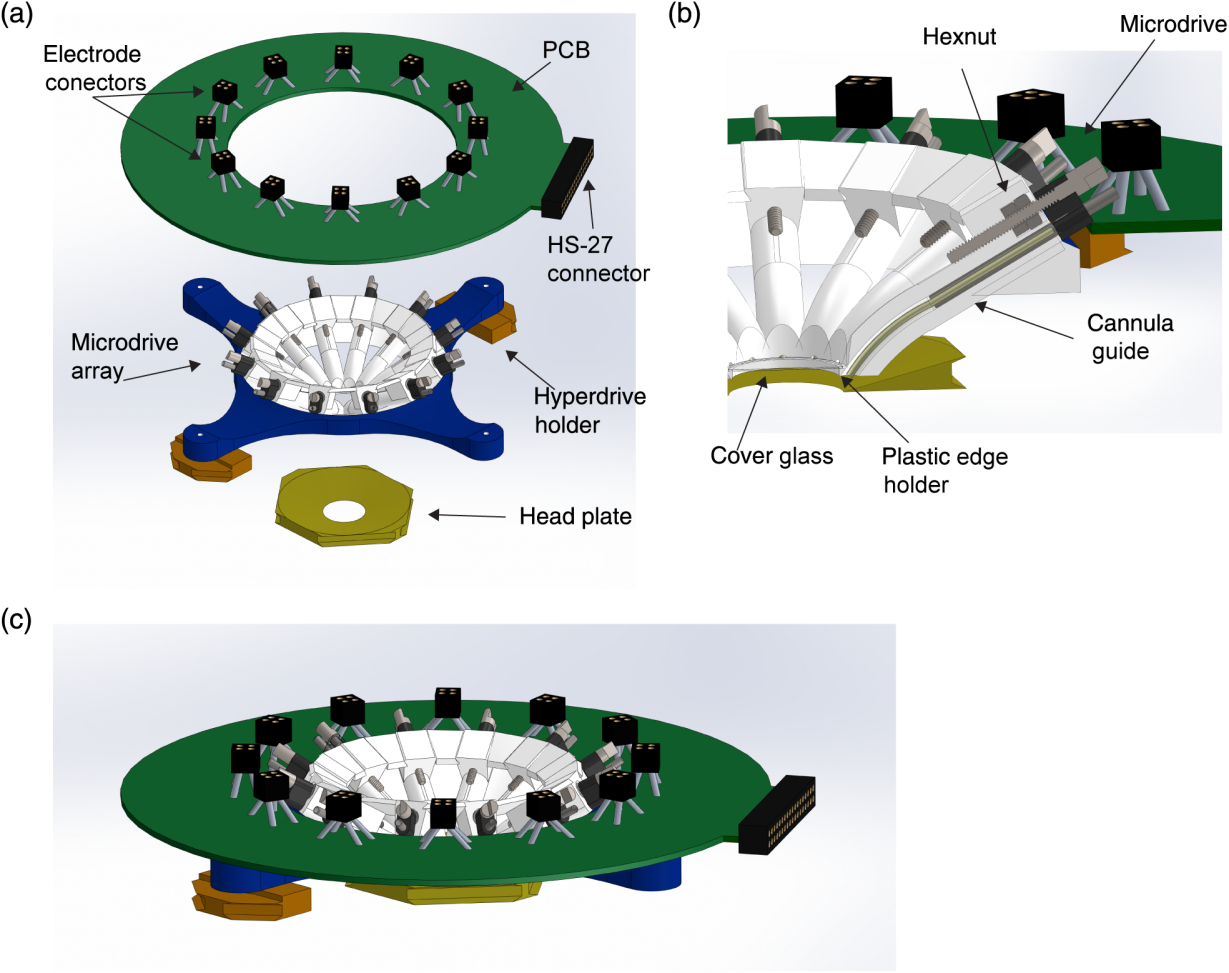
Overview of the wide-field imaging hyperdrive. (a) Renderings of the main parts of the hyperdrive: PCB; electrode connectors; HS-27 connector (Neuralynx); circular microdrive array; hyperdrive holders; and headplate. (b) Cross section of the microdrive. The metal screw moves inside the hexnuts glued into the plastic base. The tetrode (not shown) is glued to the microdrive and moves inside the guide cannula, which opens below the coverslip glass. (c) Rendering of the assembled hyperdrive.

**Fig. 2:**
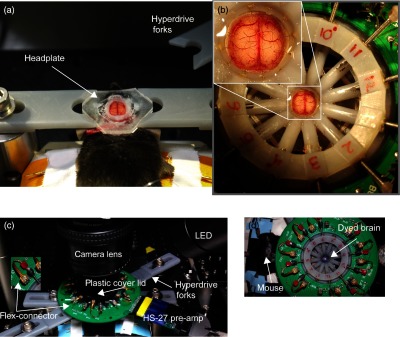
Hyperdrive and wide-field optical imaging preparation. (a) A urethane-anesthetized mouse with bilateral craniotomy is fixed between two forks using a glued-on headplate. (b) Hyperdrive centered above bilateral craniotomy. Inset: enlarged view of the bilateral craniotomy with the electrodes inserted into the brain. (c) Overview of the experimental setup. The mouse brain is covered with a cover lid. Inset: flex-connectors. (d) Top-down overview of the experimental setup when the brain has been dyed.

The setup to immobilize the animal and to support the hyperdrive consisted of a custom three-dimensional (3-D)-printed headplate with rails using acrylonitrile butadiene styrene resin [Fig. 1(a)], RIVETS™ (rodent *in vitro*/*vivo* electrophysiology targeting system), described in Ref. 43. When the animal’s head was secured between the plastic forks, the hyperdrive was centered above the headplate. Tetrodes were carefully inserted below the brain surface and traveled about 600 to 750  μm at an angle of about 45 deg. The tetrodes easily penetrated the brain surface when dura mater was removed [Fig. 2(b)] but also were able to pass through the intact dura mater. Finally, one of the tetrodes was placed just above the cortex surface to serve as a reference.

The mouse was then placed on a metal plate that could be mounted on the stage of the upright macroscope, and the skull was secured using the RIVETS™ fork system. A modified fork system was designed to hold the electrode array. The animal with the recording setup was then transferred to the experimental table and placed under the VSDI camera and over a heating pad [Fig. 2(a)].

### 2.3 Electrophysiological Recordings

We used a custom 3-D-printed plastic hyperdrive (similar in principle to the electrode array first described in Ref. 46) consisting of 12 slots for individually movable microdrive probes (Fig. 1). Each microdrive can be loaded with a tetrode, stimulating electrode, sharp metal electrode with glass/plastic coating^47^^,^^48^ or fiber optic. The hyperdrive implemented several unique features to allow for simultaneous wide-field optical imaging. The features are the following: a 7.5-mm circular opening in the center provides sufficient brain area to image [Figs. 1(a) and 2(b)]; the opening of the hyperdrive has 0.25-mm-thick rim to mount an 8-mm cover glass [Fig. 1(b)]. The cover glass stays above the tetrodes, protecting the brain surface and reducing brain pulsations;^49^ the slots for microelectrodes were made slightly curved to reduce the overall height of the hyperdrive and facilitate illumination of the brain surface for optical imaging [Fig. 1(b)]; metal hexnut glued into the plastic to provide higher precision and longevity of the hyperdrive [Fig. 1(b)].

The tetrodes were comprised of four twisted 12.5  μm nichrome wires with polyimide coating (Sandvik) and were gold plated to reduce the impedance to 100  kΩ or lower. The individual tetrode wires were soldered into a custom designed “Flex-connector” (NeuroTek) that attached to the printed circuit board (PCB) with a Mill-max connector [Fig. 2(c)]. A custom-built circuit board on the top of the hyperdrive was connected to a unity-gain headstage (HS-27, Neuralynx, Bozeman, Montana) to provide a low-noise, high impedance signal buffer [Figs. 1(c) and 2(c)]. The signal was recorded and time stamped by a Digital Lynx 16 SX system (Neuralynx, Bozeman, Montana). A reference electrode was placed above the cortex so the tip was submerged either into the brain buffer or the agarose.

To record neuronal spiking activity, the extracellular electric signal was high-pass filtered (0.1 Hz), amplified 1000 times, and digitized at 32 kHz using a Digital Lynx 16 SX system and an HS-27 headstage (Neuralynx, Bozeman, Montana). LFP traces were recorded from the same tetrodes and digitized at 32 kHz and downsampled at 312 Hz for analysis. With our setup, we were able to monitor single-unit activity for approximately half an hour (see an example in Fig. 8). To ensure we analyzed only stable units, we spike sorted experimental periods separately (e.g., spontaneous activity period, hind limb stimulation period, etc.), which lasted less than 15 min each. Spike sorting was performed semiautomatically using Klustakwik,^50^ followed by manual clustering using MClust.^51^ We only considered putative pyramidal neurons for this analysis. We selected this type of neurons by discarding fast-spiking cells based on their autocorrelogram. Histology suggests that the tetrodes tips were located in layers IV and V in the cortex (Fig. 7). Even though our setup permits the placement of electrodes at multiple depths, the electrodes that we have used have recording points at the tip only. Therefore, with the current setup, it is not possible to simultaneously record at different depths from the same location, making current source density analyses not feasible.

### 2.4 VSD Imaging

After inserting the tetrodes to the target sites, the dye RH-1691 (optical Imaging, New York, New York) was dissolved in brain buffer solution (0.5  mg/ml) and applied to the exposed cortex for 60 to 90 min. During this period, the cranial window was covered with a black plastic lid to avoid exposure of the dye to the room light [Fig. 2(c)]. After washing out unbound dye for 5 to 10 min with brain buffer solution, the brain was covered with 1.5% agarose made in HEPES-buffered saline and sealed with a glass coverslip [Fig. 2(d)]. This procedure reduced the movement artifacts produced by respiration and heartbeat. For VSD data collection, 12-bit images were captured at 150 Hz during evoked activity and at 100 Hz during spontaneous activity with a charge-coupled device camera (1M60 Pantera, Dalsa, Waterloo, Ontario) and an EPIX E8 frame grabber with XCAP 3.8 imaging software (EPIX, Inc., Buffalo Grove, Illinois). The dye was excited using a red LED (Luxeon K2, 627 nm center) and excitation filters of 630±15  nm. Images were taken through a macroscope composed of a back-to-back photographic lenses (50 mm, 1.4  f: 35 mm, 2f). This optic gives an 8.6×8.6  mm field of view, 67  μm per pixel. The excitation LEDs were driven by a custom-made power LED driver that delivers a stable constant current ranging between 0 and 700 mA. The depth of field of our imaging setup was 1 mm. Reflected VSD fluorescence was filtered using a 673- to 703-nm bandpass optical filter (Semrock, New York, New York). To reduce potential VSD signal distortion caused by the presence of large cortical blood vessels, we focused into the cortex to a depth of ∼1 mm.

### 2.5 Evoked and Spontaneous Activity

For sensory-evoked activity, we recorded 900 ms before and 4100 ms after a single 1-ms electrical pulse (300  μA) was delivered to the left hind paw for each trial. Because brain states show spontaneous fluctuations, we averaged 20 trials of stimulus presentation to reduce these effects. To correct for time-dependent changes in VSD signals due to bleaching artifact, we also collected 20 nonstimulation interleaved trials that were used for normalization of the evoked data. A 10-s interval between each sensory stimulation was used. In a previous work, VSD fluorescence was measured across the cortex using histology and demonstrated relatively high labeling at a depth of ∼750  μm.^27^ Nonetheless, to reduce regional bias in VSD signal caused by uneven dye loading or brain curvature, all VSD responses were expressed as a percentage change relative to baseline VSD responses ($\Delta F/F_{0}\times 100\%$) using MATLAB^®^ (Mathworks, Natick, Massachusetts). VSD imaging of spontaneous activity was continuously recorded in the absence of sensory stimulation for 15 min period with 10 ms (100 Hz) temporal resolution. Slow, time-dependent reductions in VSD fluorescence were corrected in MATLAB^®^ using a zero-phase lag Chebyshev bandpass filter (zero-phase filter) at 0.1 to 6 Hz. Ambient light resulting from VSD excitation (630 nm) was measured at $8.65\times 10^{-3}\;W/m^{2}$. The total duration of the VSD excitation in a typical imaging experiment ranged from 900 to 1200 s. The fluorescence changes were quantified as $(F-F_{0})/F_{0}\times 100$, where F is the fluorescence signal at any given time and $F_{0}$ is the average of fluorescence over baseline frames. To analyze the relationship between SUA and neuronal population activity, we calculated the spike-triggered average (STA) VSD (STA maps) for each neuron by taking the mean of the VSD signal over all the times when that neuron fired.^3^

### 2.6 VSDI and Electrophysiological Signals Synchronization and Comparison

VSD images and electrophysiological records were digitized on two separate acquisition systems with different sampling rates (200 Hz and 32 kHz, respectively). To synchronize these signals, we recorded the clock from the EPIX frame grabber, the excitation LED trigger, and the electrical stimulation signals in the Digital Lynx 16 SX system (Neuralynx, Bozeman, Montana) via the TTL port. During off-line analysis, we used these signals to align imaging and electrophysiological data.

We compared the LFP and VSD signals using the Pearson correlation coefficient during evoked and spontaneous activity. During evoked activity, we divided the signals into three periods: baseline, early, and late responses. Baseline activity consisted of activity before the stimulus onset (900 ms). Early evoked response consisted of the first 250 ms after stimulus onset. Late evoked responses were considered as the next 250 ms after the early evoked response. For the spontaneous activity, we calculated the similarity between LFP and VSD signals as the correlation coefficient during 15 min of spontaneous activity divided into segments of 1 s (used to calculate the mean similarity between the signals).

### 2.7 Contact for Resource Sharing

Further information and requests for resources should be directed to and will be fulfilled by the lead contact.

## 3 Results

### 3.1 Combined VSD Imaging and Multisite Electrophysiological Recording in Response to the Sensory-Evoked Stimulation

**Fig. 3:**
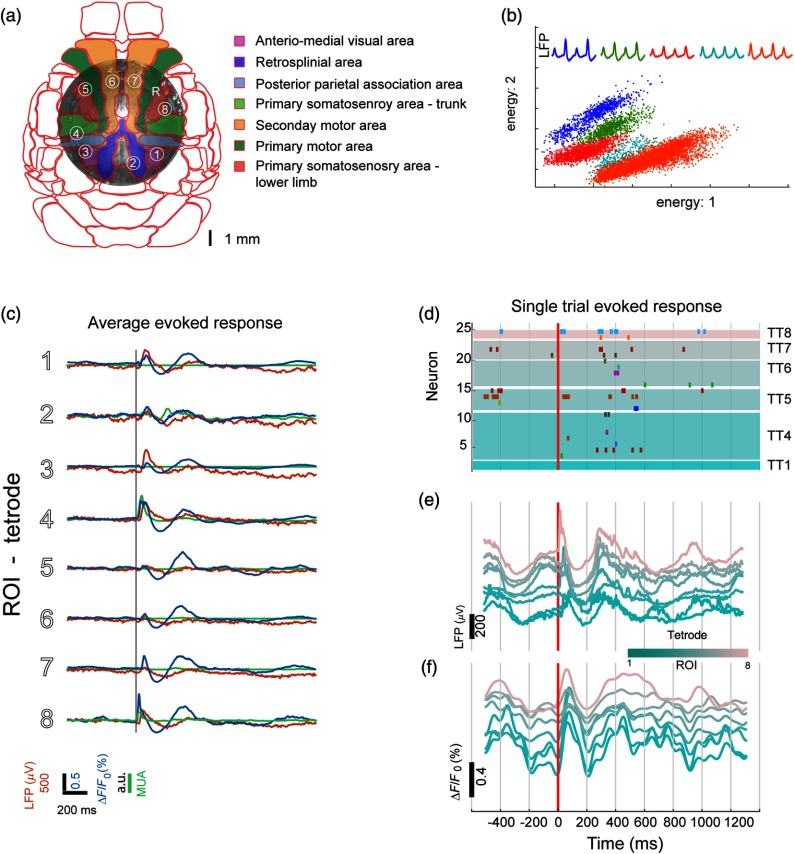
Evoked response to sensory stimulation using electrophysiology and VSDI simultaneously. (a) Photomicrograph of wide bilateral craniotomy preparation with the position of the electrodes marked as numbered circles in reference to the Allen Brain Institute Mouse Brain Atlas. (b) Example of five putative pyramidal neurons recorded in one tetrode. (c) Average evoked VSD (blue), LFP (red), and MUA (green) response to electrical stimulation of the left hind paw. The gray vertical line represents the stimulus onset. (d) Single trial raster plot of single-unit activity in response to one pulse of electrical stimulation of the left hind paw. Different colors represent different neurons. (e) LFP-evoked response to the same stimulation trial as in (d). Each line represents the LFP from one of the eight electrodes. (f) VSD-evoked response to the same stimulation trial as in (d). Each line represents the VSD signal from one of the eight ROIs in (a).

**Fig. 4:**
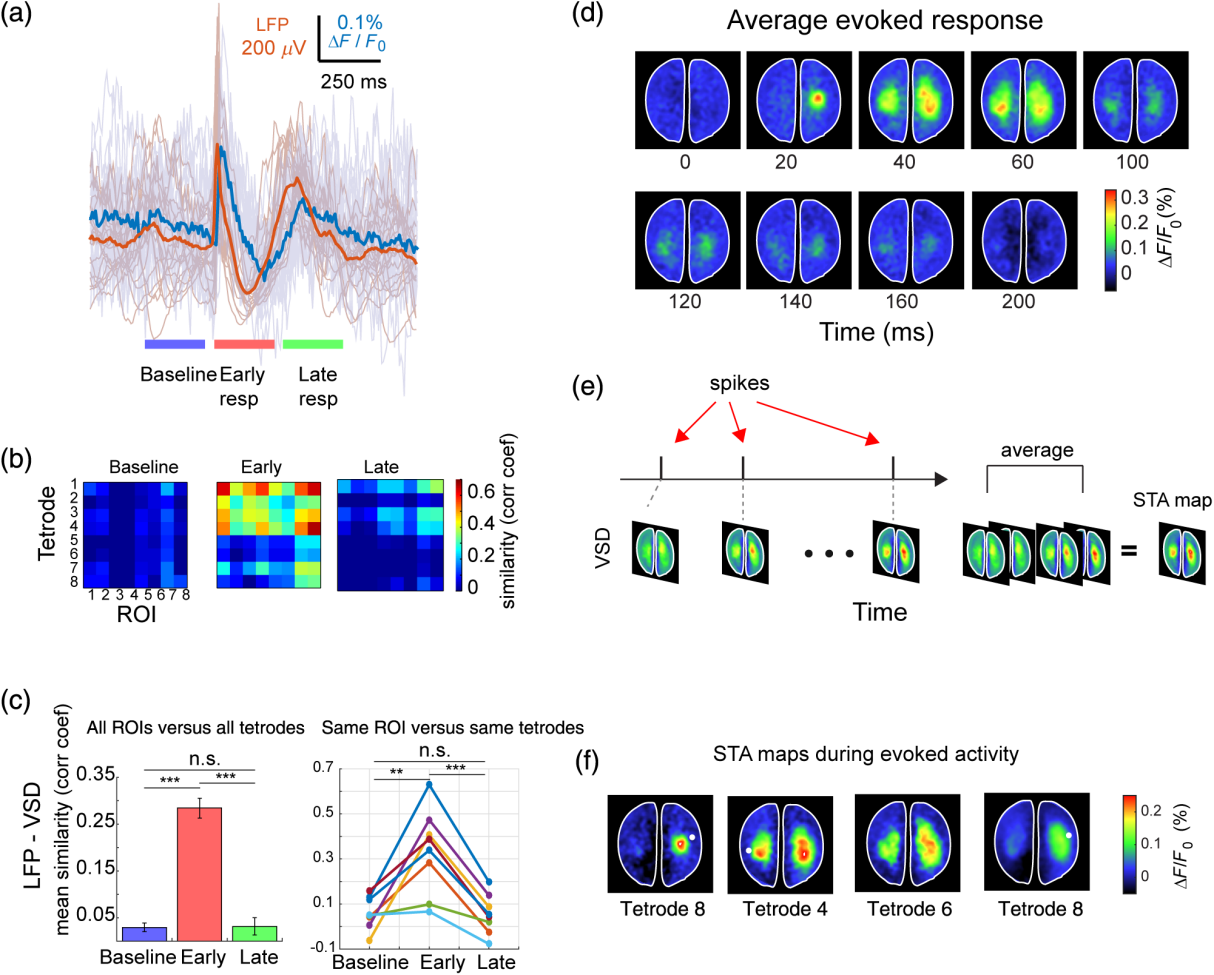
Relationship between LFP, single-unit activity, and voltage-sensitive dye signals during evoked responses. (a) Example of mean LFP (orange) from one tetrode and single trials (shaded) and mean VSD (blue) from same ROI and single trials (shaded). Evoked responses are divided into three periods: baseline (last 250 ms before stimulus onset), early evoked response (first 250 ms after stimulus onset), and late evoked response (250 ms after the evoked1 period). (b) Similarity matrices (correlation coefficient) between LFP and VSD for all tetrodes and ROIs for the three periods. (c) (Left) Mean similarity across 20 trials between LFP and VSD for the three periods for all tetrodes and all ROIs (error bars represent SEM). (c) (Right) Mean similarity across trials between LFP and VSD for the same ROIs and tetrode for the three periods. (d) Spatiotemporal pattern of the mean evoked response (20 trials) to hind paw electrical stimulation. (e) STA maps are generated as the average of the VSD frames corresponding to the times when the corresponding neuron fired. (f) Example of STA VSD maps from different neurons across the cortex. The STA maps closely resemble the evoked response in (d). The white dots represent the approximate location where the corresponding neurons were recorded.

Using a preparation with a bilateral craniotomy that exposed a large portion of the dorsal cortex in both hemispheres [Fig. 3(a)], we were able to monitor brain activity using both VSDI and electrophysiology simultaneously. To compare VSD and electrophysiological evoked responses, we averaged the VSDI signal within regions of interest (ROI) of five pixel diameter around the point where each tetrode was inserted into the cortex [Fig. 3(a)]. When stimulating the hind paw of lightly anesthetized mice (as opposed to deep anesthesia where there is almost no sensory-evoked responses. See Sec. 2), we observed unique patterns of cortical depolarization [Fig. 4(d)]. Consistent with previous studies,^22^^,^^52^^,^^53^ we found that brief electrical stimulation of left hindpaw led to activation of contralateral primary hindlimb (HL) somatosensory cortex around 20 to 30 ms after stimulus onset.

The activation of contralateral HL cortex was followed by an expansion of depolarization within the contralateral hemisphere into neighboring areas. In addition, an activation of primary HL cortex within the ipsilateral hemisphere [Fig. 4(d)] follows shortly after the initial contralateral response. The average temporal profiles of the evoked response in both VSDI and electrophysiology are similar for most of the ROIs [Fig. 3(c)]. However, for ROIs near HL somatosensory cortex (ROIs 1, 5, and 8), the latency of response is shorter than in other ROIs. However, we can observe that the evoked response is composed of two distinct periods of depolarization. This two-component conformation of the sensory-evoked response has previously been reported in the visual cortex^54^ and the somatosensory cortex.^55^^,^^56^ Figure 3(b) shows an example of sorted putative pyramidal neurons from a single tetrode. In a single trial, we can observe that evoked response to a single pulse of HL electrical stimulation expands over large areas of the cortex [Figs. 3(d)–3(f)].

To measure the similarity between LFP and VSD signals, we calculated the correlation coefficient for three periods: baseline (250 ms before stimulus onset), early evoked response (first 250 ms after stimulus onset), and late evoked response (250 ms after the early response) [Fig. 4(a)]. We calculated a similarity matrix between LFP signal from all the tetrodes and the VSD signal from all the ROIs for the three periods [Fig. 4(b)]. Note that the mean similarity between LFP and VSD signals increases significantly (t-test, p<0.001) during the first 250 ms after stimulus onset, compared to baseline for all tetrodes and ROIs [Fig. 4(c) left] and among the same tetrode and the same ROI (paired t-test, p<0.05) [Fig. 4(c) right].

Moreover, with our setup it is possible to investigate the relationship between single-unit activity and the VSD signal during particular events. For example, it has been reported that evoked responses in primary sensory areas consist of two components. The first component occurs within the first 100 ms (early) after stimulus onset and the second component occurs during 150 to 400 ms after stimulus onset (late).^54^^–^^56^ Consistent with this, we found that the evoked response in VSD, LFP signals, and SUA were formed by these two components [Figs. 3(c)–3(f) and 4(a)]. At the single-unit level, we observe that only neurons that were recorded close to the contralateral and ipsilateral HL cortical areas (neurons 4, 14, and 25, which come from ROIs 1, 5, and 8, respectively) fire within the early phase of the cortical response. However, most of the neurons recorded in the majority of remaining tetrodes participate in the late response (250 to 500 ms after stimulus onset) [Fig. 3(d)].

In order to evaluate the participation of a single neuron in the functional ensemble, we calculated the STA of VSD activity or STA maps [Fig. 4(e)]. We observe that some STA maps from neurons recorded across the cortex [Fig. 4(f)] resemble the progression of the evoked response [Fig. 4(d)] closely [compare STA maps of Fig. 4(f) and evoked response in Fig. 4(d)]. This suggests that a functional ensemble, in this case as HL-evoked response, can involve distant neurons, even outside of the corresponding sensory areas.

### 3.2 Combined VSD Imaging and Multisite Electrophysiological Recording of Spontaneous Cortical Activity

**Fig. 5:**
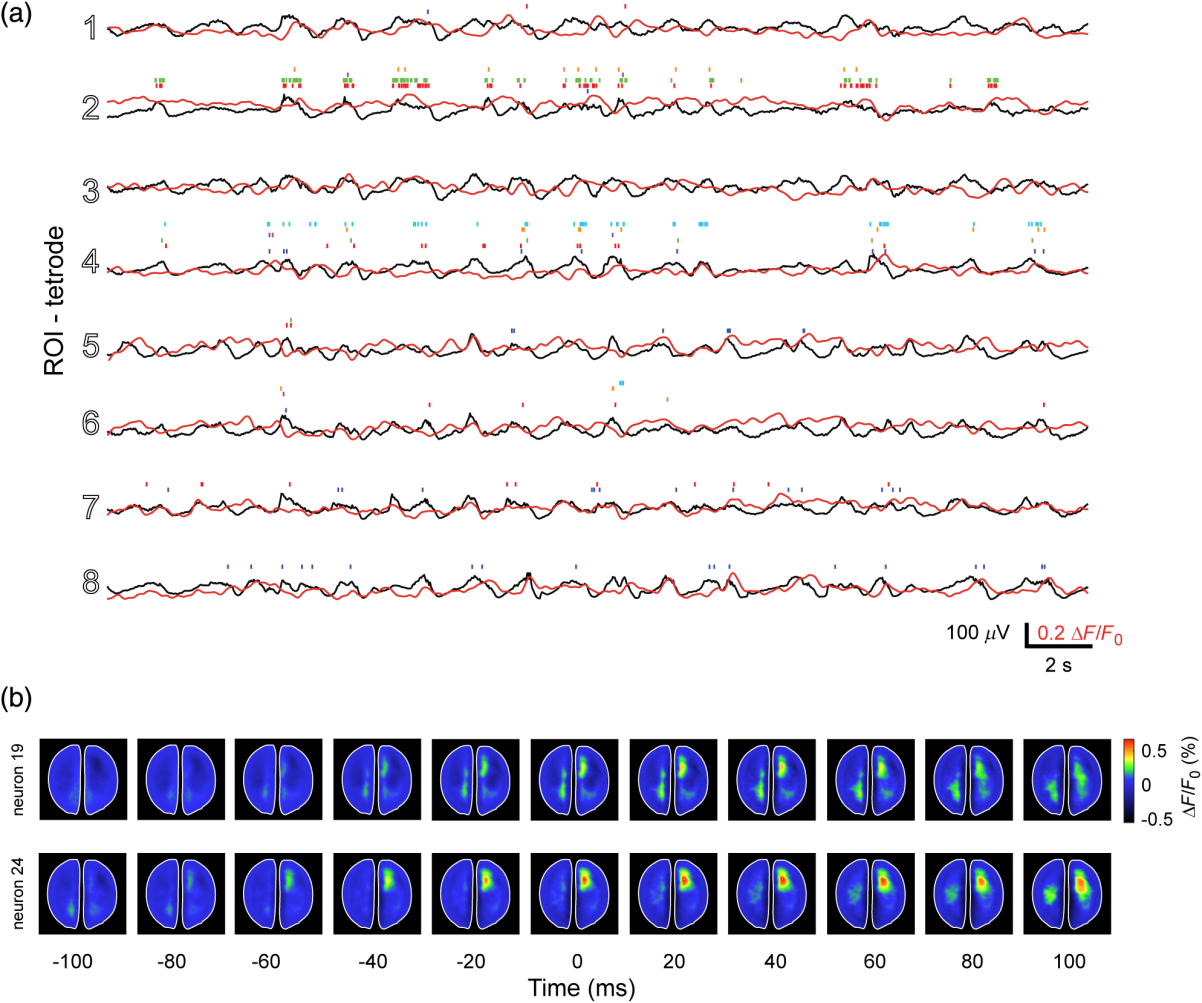
Simultaneous electrophysiological and VSD recordings during spontaneous activity in both hemispheres. (a) Simultaneous LFP, VSD, and single-unit recordings for the corresponding ROIs during 10 s of spontaneous activity. The colors of the raster plots represent different neurons recorded in the corresponding tetrode. (b) Example of STA maps for two different neurons (from different tetrodes) during spontaneous activity. The maps show the average activity 100 ms before and after the neurons fired (time 0). The white dots in the STA maps represent the approximate location where the corresponding neuron was recorded.

**Fig. 6:**
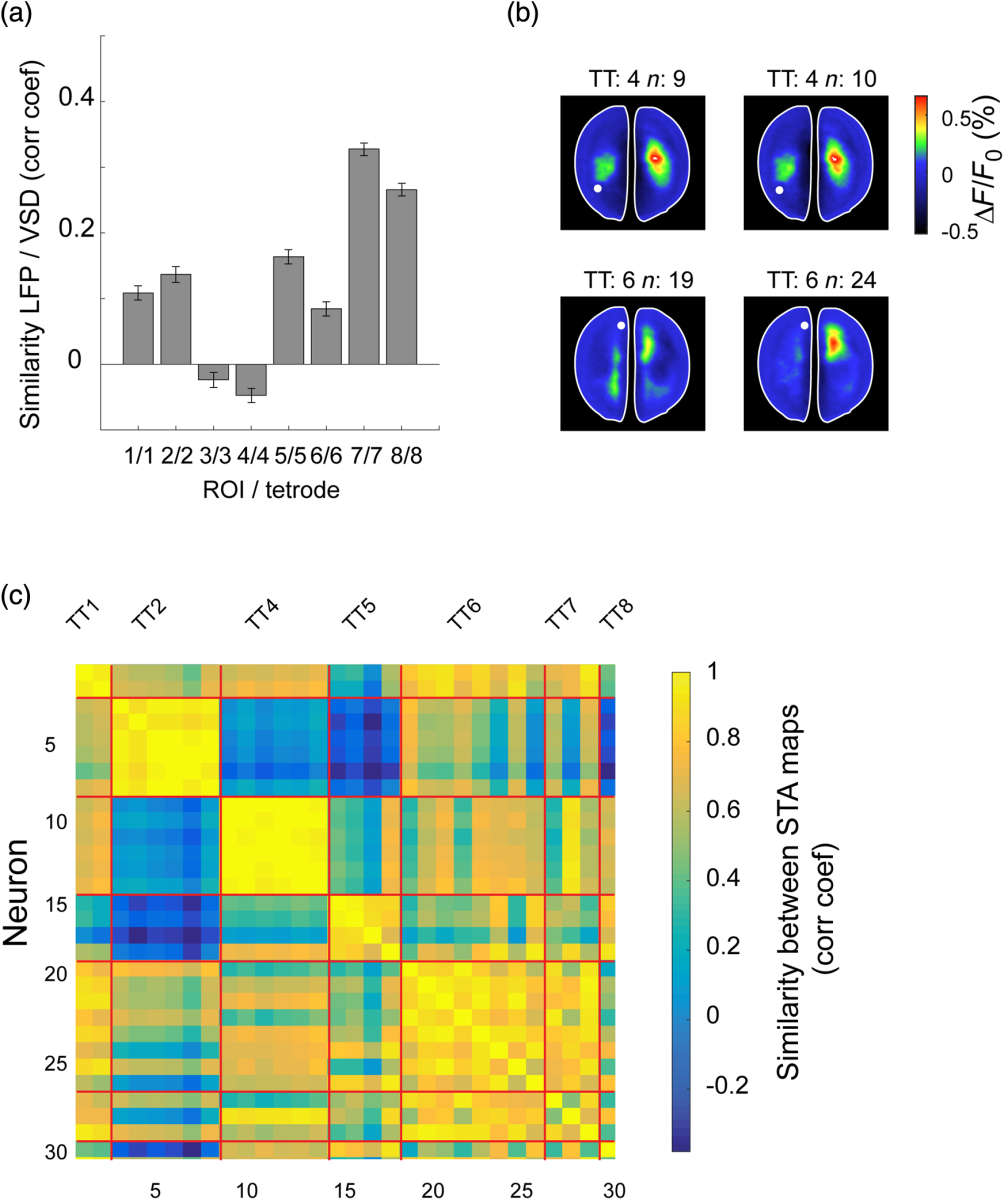
Comparison between optical and electrophysiological signals. (a) Similarity between LFP and VSD signals for all the recording locations during 15 min of spontaneous activity (for 1 s segments). (b) STA maps of two neurons recorded in the same tetrode (TT), which are very similar (top row) and STA maps of two neurons recorded in the same tetrode, which are different (bottom row). (c) Similarity matrix (correlation coefficient) for the STA maps generated for all neurons.

**Fig. 7:**
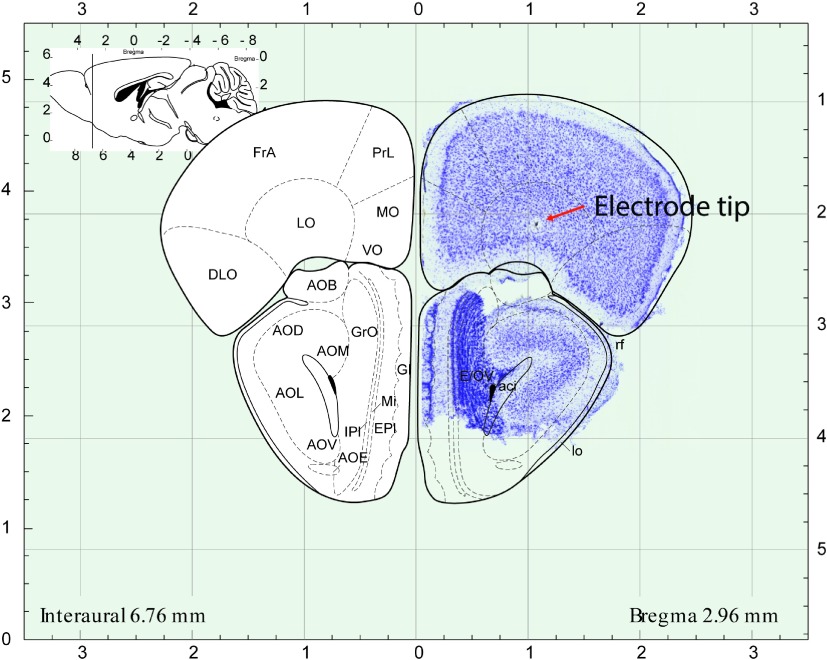
Histological analysis of electrode sites. Diagram of the coronal section (left) of the mouse brain atlas^81^ and representative coronal section stained with cresyl violet with marked electrode tip (arrow). FrA, frontal association cortex; PrL, prelimbic cortex; MO, medial orbital cortex; VO, ventral orbital cortex; LO, lateral obrital cortex; DLO, dorsolateral cortex; AOD, anterior olfactory nucleus dorsal; AOL, anteior olfactory nucleus lateral; AOM, anterior olfactory nucleus medial; and AOV, anterior olfactory nucleus ventral.

**Fig. 8:**
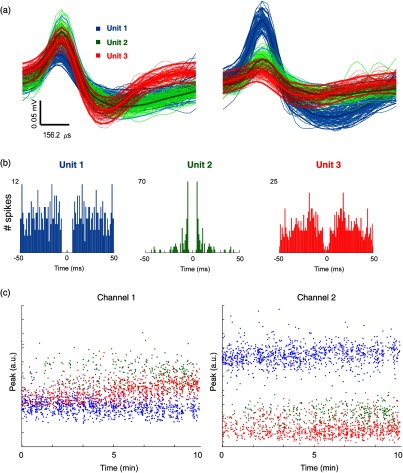
Waveforms and stability of recorded units. (a) Examples of one hundred randomly selected waveforms from a 10 min recording in two recording channels (left and right). Bold lines indicate mean and standard deviation for the corresponding waveforms. (b) Autocorrelogram of the same units. Percentage of the spikes within 2 ms interspike interval window was 0% for units 1 and 2, and 0.22% for unit 3. Note a prominent bursting activity in unit 2. (c) Peak amplitude of all the recorded spikes for these three units during a 10 min recording. Based on the waveforms and autocorrelograms, units 1 and 2 can be classified as putative excitatory neurons and unit 3 as a putative interneuron.

The brain is constantly active, even in the absence of sensory input or motor output.^57^ To evaluate the differences between the two signals, we compared the LFP and the VSD signal when there was no stimulation. In general, VSD and LFP signals show similar dynamics as is observed in Fig. 5(a). However, this similarity varies with time and cortical location. This type of variability has previously been reported for small regions of visual areas during spontaneous activity.^58^ However, with our setup, it is possible to extend this comparison to wider regions of the brain. We measured the similarity between LFP and VSD signal for the same locations (tetrode and ROI) for 1 s periods for 15 min of spontaneous activity. We observed that both signals have different levels of similarity depending on the recording location [Fig. 6(a)]. We notice that there is a high correlation among most of the STA maps. However, surprisingly, for tetrodes 3 and 4 the correlation is close to zero. A potential explanation for the low correlation for tetrodes 3 and 4 could be a different depth than the rest of the tetrodes.

In addition to comparing the LFP and VSD signals, with our setup, it is possible to evaluate the participation of individual units from distant regions in cortical ensembles (STA maps). Previously, the relationship between VSD (or wide-field imaging signals) and SUA has been studied by monitoring neurons from small regions of the brain.^3^^,^^58^ With our setup, it is possible to study such relationship over large and distant areas. We observed that in most cases the STA maps are similar for the neurons recorded in the same tetrode [Fig. 6(b) top row]; however, there are cases in which one can see different maps in the same tetrode [Fig. 6(b) bottom row]. This suggests that there are neighboring neurons potentially participating in different “cortical ensembles.” In order to quantify the similarity between the STA maps of all recorded neurons during spontaneous activity, we calculated the correlation matrix in Fig. 6(c). We observe that there are regions in the cortex where the neurons participate in similar patterns of activity more than others (e.g., TT2 and TT6).

Finally, we compare two STA maps for different neurons (recorded in different tetrodes) before and after each neuron fired [Fig. 5(b)]. We observe that, in the top row case, on average, the neuron fires when a large population activity increases (time 0). In contrast, in the bottom row, the neuron fires after the large population activity starts to increase (time −20  ms). This demonstrates that there is potentially a different pattern of cortical activity that might be related to different spiking patterns of different neurons.^59^

## 4 Discussion

Electrode arrays of different configurations have been widely and successfully used in neuroscience studies. Although these studies have greatly improved our understanding of cortical dynamics, their conclusions are limited due to the poor spatial coverage that causes a difficulty in monitoring neuronal activity across large cortical areas. In addition, the intrinsic limitation of electrophysiological signals in source localization due to volume conductance makes it difficult to study contributions of particular cell types or brain regions.^14^^,^^60^^,^^61^ Conversely, recent advances in protein-based activity indicators such as voltage,^62^^–^^64^ calcium,^65^^,^^66^ and glutamate^67^ sensors make it possible to target-specific type of neurons or locations in the brain. Such development in brain imaging technology has made brain imaging an extremely useful tool to monitor neuronal populations at a mesoscale level. Therefore, the combination of the multiple-site electrophysiology and VSDI represents a great opportunity to study brain function at different scales simultaneously.

However, recording large neuronal population activity using a large number of electrodes is rarely compatible with optical imaging due to technical difficulties. On one hand, the need to provide independent movement and wire routing for each electrode or tetrode inevitably makes an electrode array bulky. On the other hand, wide-field imaging requires a large cranial window that needs to leave a clear space for excitation and imaging. As a way to resolve these issues, we developed a type of electrode array to combine deep cortical recordings with wide-field optical imaging (Figs. 3 and 4). The array provides multisite electrophysiological recordings with arbitrary depth and a choice of electrodes to be used without interfering with wide-field imaging.

In this paper, we demonstrate the advantage of using our setup by simultaneously recording VSDI and electrophysiological data. With this method, it is possible to analyze the relationship between electrophysiological signals and VSDI recordings at different brain locations. In particular, we showed that, on average, the temporal dynamics between LFP- and VSDI-evoked responses are similar even for distant regions in the cortex (Fig. 4). Analogously, we showed that during spontaneous activity the LFP and VSD signals are similar during 1 s periods (Fig. 5). Moreover, we showed that neurons that were recorded close to the HL S1 area (tetrode 5) participate in activity patterns that resemble the average HL-evoked pattern [top row STA map in Fig. 4(f)]. However, even the neurons recorded far from the HL S1 area (tetrode 7), participate in population activity that vaguely resembles the HL-evoked activity pattern [bottom row STA map in Fig. 4(f)].

This result highlights the advantage of combining multiple-site tetrode recordings and VSDI. Similarly, we demonstrated that, even though in most of the cases neurons recorded in the same location participate in a similar functional ensemble, there are cases where neighbour neurons (recorded in the same tetrode) are involved in different cortical networks [Fig. 6(a)]. However, our study was limited to a single brain depth so for an analysis of propagation of activity at different cortical layers, different electrode probes would be needed.^35^ Finally, we showed that the spatiotemporal dynamics of cortical activation patterns can be distinct even for neurons recorded in the tetrode [Fig. 6(c)]. This result demonstrates that spiking activity at single-cell level can be related to different neuronal ensembles at a population level, which is reflected in the STA maps.^59^^,^^68^ Further analysis using this approach could potentially clarify the relationship between cell assemblies (i.e., sequential activation of distinct neurons) at a local population level and cortical processing at a more global scale using wide-field imaging. Such relationships play an important role in top-down sensory processing,^55^^,^^69^^,^^70^ in memory, where sharp-wave events at a local hippocampal circuit might be related to more global cortical activity^16^ or in cortical processing in general, as a means to quantify neuronal “population coupling” or “packet-based communication.”^1^^,^^68^ An interesting future research avenue that is possible to study with our setup is precisely the combination of recordings of subcortical structures such as the hippocampal formation or/and the thalamus to expand the current understanding of the interaction of such brain regions with the cortex at the mesoscale level. Targeting the subcortical structures would require adjustments to make the angle at which the probe enters the brain steeper. However, this can be easily done considering that this part of the setup is custom 3-D printed.

In our current method to combine VSDI and electrophysiology, there are important issues to note. We found that brain damage due to electrode loading inevitably leads to bleeding, which can compromise the quality of VSD imaging. Having dura mater removed permits easy rinsing of brain surface, whereas in dura-intact preparations the extravasations may localize in subdural space. Among the electrodes we used in our experiments, we found the conventional tetrodes to be the most user-friendly. They are inexpensive, easy to make, and durable. Conversely, sharp metal electrodes^47^ have the advantage of producing less damage to the tissue, but they are considerably more fragile than conventional tetrodes. Our setup could be easily adapted to be used with Uwe Thomas tetrodes,^71^ flexible silicon probes,^72^ or optical fiber probes for optogenetic stimulation or imaging.^73^^,^^74^ The latter may be used for local optogenetic stimulation or imaging for further investigation of local and distributed neural circuits. One limitation of the present study is that we only recorded acutely from urethane-anesthetized mice due to the toxicity of VSD. It would be interesting to expand our setup for chronic recordings in anesthetized or awake animals. In principle, this is possible with some modifications to our setup.

With the development of genetically modified mice that express a variety of protein-based indicators (e.g., voltage,^62^^,^^63^^,^^64^ calcium,^65^^,^^66^ or glutamate^67^) in different brain cells and the development of soft cranial windows for chronic imaging,^75^^,^^76^ it is possible to do multiple electrode insertions for long-term studies. However, our setup was designed specifically for acute recordings in mice and such modification for chronic recordings in awake animals would require considerable changes. Yet, with our setup it is possible to record in head-fixed awake animals in a postanesthesia preparation if a different anesthetic such as isoflurane is used instead of urethane.^77^

In summary, we present a method to combine electrophysiological and wide-field imaging simultaneously. Such combination of techniques allows monitoring the brain activity at different scales with high temporal resolution over large brain areas, and it represents a great tool to study brain function in general.^19^^,^^20^^,^^78^^–^^80^ Therefore, the methodology presented in this paper further expands the available tools to improve the current understanding of brain function.

## Appendix

Once brain activity was recorded, we passed an electrical DC current (200 mA) from the electrodes to the animal tail for 10 sec to cause a lesion that allowed us to locate the position of the electrode tips (see Methods Sec. 2). Figure 7 shows an example of an electrode located in the orbital cortex using cresyl violet.

With our setup we were able to record single-unit activity at multiple brain regions and VSD imaging over large areas in both hemispheres. Figure 8 contains examples of different types of neurons recorded. In particular, we show one hundred randomly selected waveforms for three different neurons recorded in the same tetrode. In addition, we also show the stability of these three neurons for a 10-min period.
